# St. Bartholomew's Hospital

**Published:** 1906-02-24

**Authors:** 


### ST. BARTHOLOMEW'S HOSPITAL.

Lord Ludlow, Treasurer of St. Bartholomew's Hospital, in issuing an appeal on behalf of that institution, says :—

May I call public attention to the want of funds for the rebuilding of St. Bartholomew's Hospital?

The greater part of the existing buildings were erected over 150 years ago, and the need of reconstruction to render them adequate to the present day requirements will, I think, be apparent.

Having regard to the very large sum that would be required to carry out the complete scheme, the governors decided to undertake part only at a time, as funds were forthcoming.

The first block, which comprises general and special departments for out-patients, a casualty department, a dispensary, quarters for the resident medical staff, hospital kitchens, and other domestic offices, is now in course of erection, and the greater part of the cost (£120,000) must be met by the end of the current year.

The governors have received generous response to their appeal for assistance, but a sum of £20,000 is still urgently needed to complete this block. The accommodation provided therein is most seriously required in order that the work of this great hospital may be efficiently carried on, and I most earnestly appeal to the benevolent public for contributions towards this object.

